# Temperature Gradient Measurements by Using Thermoelectric Effect in CNTs-Silicone Adhesive Composite

**DOI:** 10.1371/journal.pone.0095287

**Published:** 2014-04-18

**Authors:** Muhammad Tariq Saeed Chani, Kh. S. Karimov, Abdullah M. Asiri, Nisar Ahmed, Muhammad Mehran Bashir, Sher Bahadar Khan, Malik Abdul Rub, Naved Azum

**Affiliations:** 1 Center of Excellence for Advanced Materials Research (CEAMR), King Abdulaziz University, Jeddah, Saudi Arabia; 2 Department of Chemistry, Faculty of Science, King Abdulaziz University, Jeddah, Saudi Arabia; 3 GIK Institute of Engineering Science and Technology, Topi, Swabi, Khyber Pakhtunkhwa, Pakistan; 4 Physical Technical Institute of Academy of Sciences, Dushanbe, Tajikistan; RMIT University, Australia

## Abstract

This work presents the fabrication and investigation of thermoelectric cells based on composite of carbon nanotubes (CNT) and silicone adhesive. The composite contains CNT and silicon adhesive 1∶1 by weight. The current-voltage characteristics and dependences of voltage, current and Seebeck coefficient on the temperature gradient of cell were studied. It was observed that with increase in temperature gradient the open circuit voltage, short circuit current and the Seebeck coefficient of the cells increase. Approximately 7 times increase in temperature gradient increases the open circuit voltage and short circuit current up to 40 and 5 times, respectively. The simulation of experimental results is also carried out; the simulated results are well matched with experimental results.

## Introduction

With the expanding human population, the demand for energy is increasing continuously, and the sustainable energy supply is arising as one of major problems of 21^st^ century. By the year 2050, the expected world population will be 10.6 billion and the energy demand will also become double because of industrialization of societies and better life standard. But, the currently serving fossil fuel resources are limited and causing global warming and environmental issues, so, the scientific society is trying to explore environmental friendly energy resources. Now, the efficient production of clean and sustainable energy is the most provoking challenge of coming few decades [1]–[4]. To meet the future energy challenges, the thermoelectric phenomena can play an important role, which involve the conversion of heat to electricity and endow with the methods for materials heating and cooling [1]. The sustainability of the electricity can also be improved by scavenging of waste heat from industrial processes, factories, power plants, automotive exhaust computers, home heating and even from the human body by the use of thermoelectric generators [4]–[6].

Use of waste heat for the generation of electric power is of prime importance to meet the world's future energy requirements [7]. The devices that are used for the conversion of heat energy into electricity are the semiconductor thermoelectric cells, which are also used for the cooling at thermoelectric refrigerators. Thermoelectric cells work on the principle of Seebeck effect [8] and their efficiency (Z) is determined by the following expression [9]:
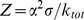
(1)where α and σ are the Seebeck coefficient and electrical conductivity, respectively, while k_tot_ is the total thermal conductivity, which is equal to sum of the electron (k_el_) and phonon (k_ph_) thermal conductivities. The increase in efficiency of thermoelectric generators depends, first of all on decrease in phonon thermal conductivity (k_ph_). In this way, the layered chalcogenides with complex crystal structure are investigated intensively [10]. During the last years, thermoelectric cells based on 11 µm thick layers of n-Si/SiGe-p-B_4_C/B_9_C deposited on the silicon substrate has been fabricated, which showed high efficiency of 15% [11]. At the same time the thermoelectric effect is used not only for the conversion of energy, but also for the measurements of temperature gradient in instrumentation, which in turn is used for the measurement of concentration of gases (like CO, CH_4_ and C_2_H_5_OH,etc) [12] by the use of thermoelectric cells on the base of oxides of tin and indium. In ref. [13] the Bi_2_Te_3_–Sb_2_Te_3_ (p-type) and Bi_2_Te_3_–Bi_2_Se_3_ (n-type) based thermoelectric cells for the measurement of temperature gradient are shown. It is also reported that these cells have high thermoelectric figure of merit (ZT) and can be used to determine the velocities of gas flow.

In addition to chalcogenides, the transition metal oxides are also very attractive thermoelectric materials. These materials have excellent mechanical, chemical and electronic properties along with fascinating thermoelectric characteristics like tunable phonon and electronic transport properties, high electrical conductivity and Seebeck coefficient, high temperature stability and well-known synthesis processes. Some representative thermoelectric metal oxides are the MnO_2_, TiO_2_, ZnO and WO_3_ [10], [14]–[16]. Recently, Walia et al. implemented ZnO and MnO_2_ for the fabrication of wave-based thermo-power energy generation devices; the concept of thermo-power waves demonstrates great potential for the miniaturization of power sources by maintaining their capabilities of energy generation. These devices have been fabricated by sequential deposition of thermoelectric material (ZnO or MnO_2_) and solid fuel (nitrocellulose) on Al_2_O_3_ substrate. The thermo-power waves are generated by solid fuel's exothermic reaction and then propagated through thermoelectric material. This self propagation of waves resulted in very high output voltage of 500 mV and 1.8 V in case of ZnO and MnO_2_ based devices, respectively, while, their corresponding room temperature Seebeck coefficients are −360 µVK^−1^ and −460 µVK^−1^ [15], [16].

Presently, not only inorganic but also organic materials based thermoelectric sensors and generators are investigated on the base of Seebeck effect. Sumino et al. [17] investigated the properties of organic thin film thermoelectric cells based on semiconductive bi-layer structures in which C_60_ and Cs_2_CO_3_ were used as n-type elements, while, pentacene and F_4_-TCNQ (tetracyanoquinodimethane) as a p-type elements. It is reported that the Seebeck coefficient for n-type and p-type elements was respectively measured as 0.19 and 0.39 mV/°C and it is also concluded that the bi-layer structures allow to increase conductivity and efficiency of the thermoelectric cells.

Investigations on the thermoelectric properties of the nano-materials show that their Seebeck coefficient and ZT as a rule are higher than that of traditional thermoelectric materials [18]: for example, maximum figure of merit of nano-BiSbTe and BiSbTe approximately were equal to 1.5 and 1.0. Theoretical investigations show that the ZT of CNT based thermoelectric cells can be larger than 2 [19], [20]. However, experimental results show that ZT is in the range of 10^−3^ to 10^−2^, Seebeck coefficient is around of 40 µV/°C (at room temperature) [20]. The value of ZT can be increased up to 0.4 by plasma treatment of CNT in argon atmosphere, which causes to increase Seebeck coefficient and decrease thermal conductivity of the material. Nevertheless the figure of merit is low for utilization of the CNT for conversion of the heat energy into electricity [20]. The thermoelectric properties of ultra small single-wall carbon nanotubes showed that ZT can be increased by surface design, formation of bundles, increasing the tube length, and so on, which significantly reduce the phonon and electron-derived thermal conductance [21].

Investigation of thermoelectric properties of single-wall carbon nanotube/ceramic nanocomposites (3Y-TZP/Al_2_O_3_) produced by spark-plasma-sintering showed that the thermoelectric power and ZT of the composites increase with increase in temperature. The thermoelectric power changed from 28.5 µV/K to 50.4 µV/K on change in temperature from 345 K to 644 K, while the value of ZT is ∼0.02 at 850 K, which is double than that of SWCNT (in pure form). These factors indicate the potential of CNTS for their use as a thermoelectric material [22]. Seebeck coefficient increased linearly with increase of temperature. The conductivity of the composite decreased with temperature showing the metallic behavior. The effect of single (SWCNTs), few (FWCNTs) and multi-walled CNTs (MWCNTs) on the thermoelectric performance of CNT/polymer (Nafion) nanocomposites was studied by Choi et al. [7]. It was found that the electrical properties of the CNT/Nafion nanocomposites were primarily affected by the CNTs since the Nafion acts as an electrically non-conducting matrix. The thermal conductivity of the nanocomposites was dominated by the Nafion mainly due to weak van der Waals interaction. The electrical conductivity and Seebeck coefficient increased as the concentration of CNTs was increased. It was found that for thermoelectric applications FWCNTs and MWCNTs are preferred over SWCNTs in CNT/Nafion nanocomposites. In polyaniline/carbon nanotube (PANI/CNT) composites in which PANI coats CNT networks, the enhanced Seebeck coefficients and figure of merits were obtained [23]. It was found that the thermoelectric parameters are several times larger than those of either of the individual components. It is also considered that this new approach has potential for synthesizing high-performance thermoelectric materials. Therefore, it would be reasonable to investigate the possibilities to use CNTs in the thermoelectric cells because of their relatively low cost and commercial availability. In this paper, the results of the investigation of thermoelectric cells fabricated on the base of CNT-silicone adhesive composites are presented.

## Experimental

**Figure 1:**
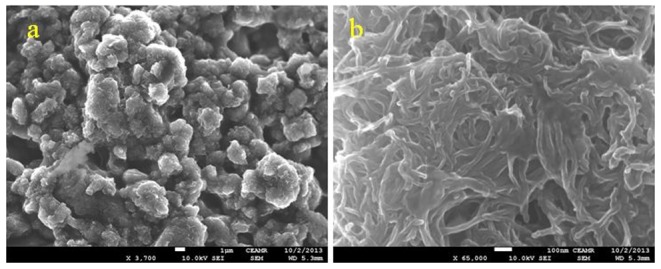
SEM image of the CNT-silicone adhesive composite layer at lower (a) and higher (b) magnifications.

Commercially available (Sun Nanotech Co Ltd., China) multi-walled carbon nanotubes (MWNTs) powder and liquid silicon adhesives (Hero Gum) were used for the fabrication of composites. The diameter of MWNTs varied between 10–30 nm. For the fabrication of thermoelectric cells, the composite was prepared by mixing the multi-walled carbon nanotubes powder with silicone adhesive. The ratio of components was 1∶1 by weight. The medical glass slides were used as substrate. Before deposition of composite layer on glass substrates, the substrates were cleaned by methanol and dried. The layers of composite were deposited by sequential use of drop-casting and doctor blade technologies. The length, width and total thickness of the composites layers were equal to 45 mm, 10 mm and 100 µm respectively. The thickness of the CNT composite layers was controlled by screen and measured by optical microscope. After deposition, the samples were dried for one day in room temperature conditions and then for 2hrs at 90 °C. Figure 1 shows SEM image of the CNT-silicone adhesive composite layer at various magnification. The composite layer consisted of grains, which are in the range of 1 to 4 micron.

**Figure 2:**
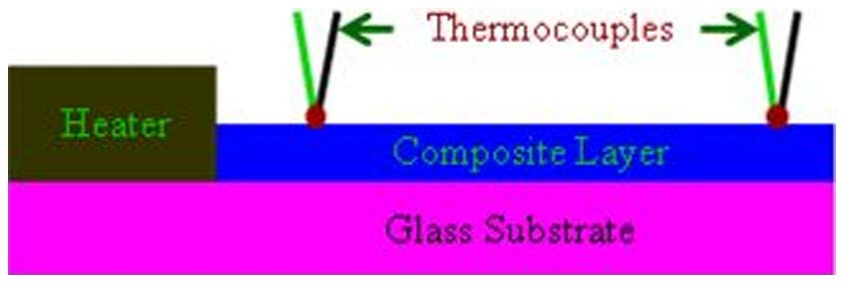
Schematic diagram of the thermoelectric cell based on CNT-silicone adhesive composite.

For the measurement of temperature, the thermocouples were used, which also played the role of electrodes as well for the measurement of voltage. The thermocouples were fixed at the cell's surface by silver paste. Figure 2 shows schematic diagram of the thermoelectric cell. The temperature, voltage and current were measurement by using FLUKE 87 multimeter, while, the Seebeck coefficients were obtained as a ratio of the voltage developed between “hot” and “cold” thermocouples (Fig. 2) and temperature gradient (ΔT).

## Results and Discussion

**Figure 3:**
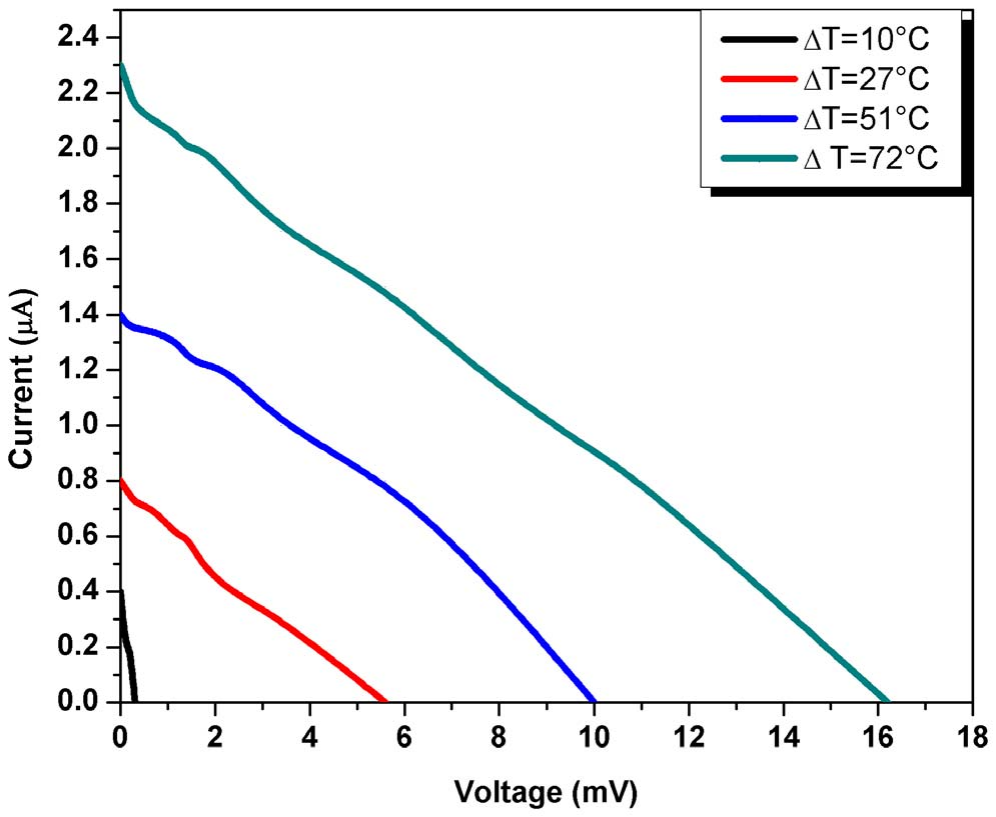
Current-voltage characteristics of the thermoelectric cell at different values of temperature gradient (ΔT).

**Figure 4:**
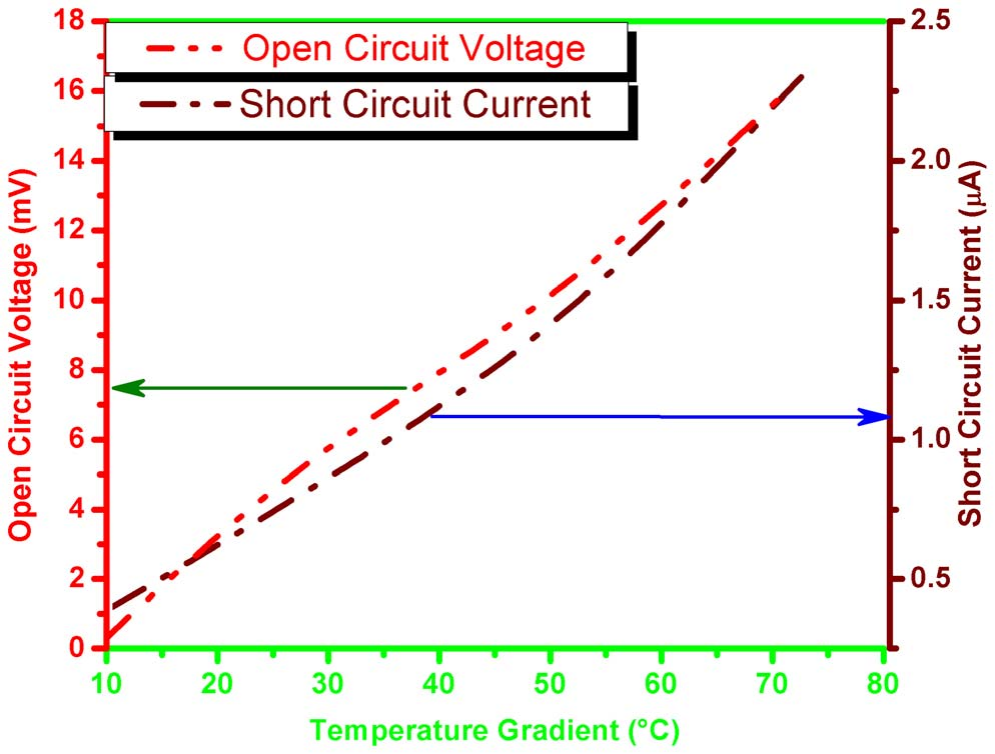
Open-circuit voltage and short circuit current- temperature gradient relationships for the thermoelectric cell based on CNT-silicone adhesive composite.

**Figure 5:**
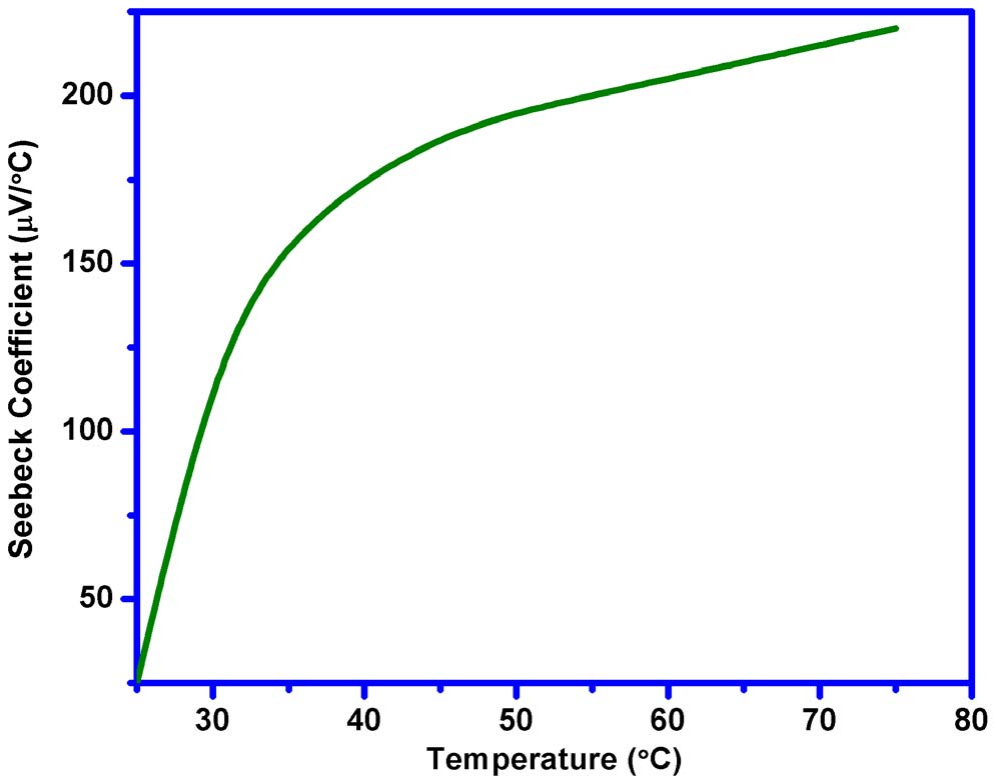
Seebeck coefficient (α)-temperature relationship of the cell.

Figure 3 shows current-voltage characteristics of the thermoelectric cell at different values of temperature gradient (ΔT) and at different values of load resistance of the cell. It can be seen that as ΔT increases the open circuit voltages and short circuit currents of the cell also increase. Figure 4 shows open-circuit voltage and short circuit current-temperature gradient relationships. These relationships are quasi-linear, that make application of the cells more suitable. As the temperature gradient increases approximately 7 times, the open circuit voltage and short circuit current increase up to 40 and 5 times respectively. The Seebeck coefficient (α)-temperature relationship is shown in Fig. 5. It can be seen that initially α increases with temperature and shows saturation behavior. Usually, the CNT composite samples are the blend of semiconductor and metallic phases. The temperature dependence of the Seebeck coefficient of the CNTs can be described by the following expression [24]

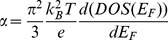
(2)where T is temperature, DOS(E_F_) is density of states on the Fermi energy level (E_F_). In the case of metallic and semiconductive nanotubes, the derivative d(DOS(E_F_))/dE_F_ is equal to zero or non-equal, respectively [24]. In the case of the metallic or degenerate semiconductor behavior of the CNTs, the following expression may be more relevant [4], [15], [25]:
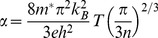
(3)where *n* and *m* ^*^ are the carrier concentration and the effective mass of the carriers, respectively. It means that metallic behavior of the investigated CNT composite observed in Seebeck coefficient (α)-temperature relationship shown in Fig. 5 shows that “metallic phase” is dominating over of “semiconducting phase” in this case. Under the temperature gradient, the measurements of the polarity of open-circuit voltage, in particular, positive potential of the “cold” side of the sample with respect of the “hot” side revealed that the CNT-silicone adhesive composite is a p-type material.

Previously, it was stated that limitation in the sizes of particles led to the increase in efficiency of the thermoelectric cells. The cells fabricated on the base of nanomaterials were more efficient as compared to the ordinary materials of the same composition. However, it was observed that in the case of intrinsic undoped-nano-materials, the thermoelectric effect was relatively low due to symmetrical contribution of the electrons and holes [18]. Therefore, due to the increase in thermoelectric parameters like figure of merit and efficiency, the practical application of the nano-materials may be realized by doping them with n-type and p-type impurities.

As the electronic properties are concerned, it is well known that CNTs show metallic or semi-conductive (small band gap) behavior depending on the orientation of the graphene lattice with respect of CNT axis [26]. This idea is also supported by the resistance temperature relationships investigated by us: it is found that with increase in temperature from 29°C to 72°C, the resistance of the cells decreases from 662 Ω to 586 Ω, i.e. temperature coefficient of the resistance is equal to −0.27%/°C.

The conductivity of charges in the composite can be attributed to percolation theory [27], [28]. According to this theory, the average conductivity can be calculated using this expression:
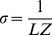
(4)where *L* is the characteristic length between sites and *Z* is the average resistance of the connecting path between sites. Here total conduction can be considered as a conduction of both layers. Probably, the contribution of the CNT layer is considerably larger, as the change in the thickness of the CNT layer and concentration of CNT influences much to the total conduction of the composite.

The adhesive in the composite plays a vital role in making the CNT layers firm, so that these layers could provide stable properties. This effect was practically observed during the current study. Another expected role of adhesive is that it may affect the thermoelectric properties of the composite. But, the comparison of the Seebeck coefficients of the investigated composite with different CNTs and CNT-composites [7], [20]–[23] shows that the effect of adhesive to the thermoelectric properties was really negligible as the value of the main parameter, Seebeck coefficient, measured by us was in the similar range as presented in the relevant references.

Unlike to thermoelectric generators, where the efficiency or figure of merit are most important parameters, the most important parameters for the temperature gradient sensors are the Seebeck coefficient, linearity of the voltage-temperature gradient and the range of the temperature gradient. In principle, both organic or inorganic semiconductors can be used for the temperature gradient sensors. For example, as organic semiconductors the quasi-one dimensional crystals of tetracyanoquino-dimethane complexes could be used, where the Seebeck coefficient is around of 1000 µV/°C [29]. At the same time, for practical applications, the growth of sufficiently large sized crystals is difficult. Therefore, utilization of the thin film thermoelectric cells on the base of the CNT composites seems reasonable. During last few years, the properties of thin films based cells have been improved [18].

The simulation of current-voltage behavior is carried out by using the following mathematical relationship [30]

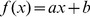
(5)


**Figure 6:**
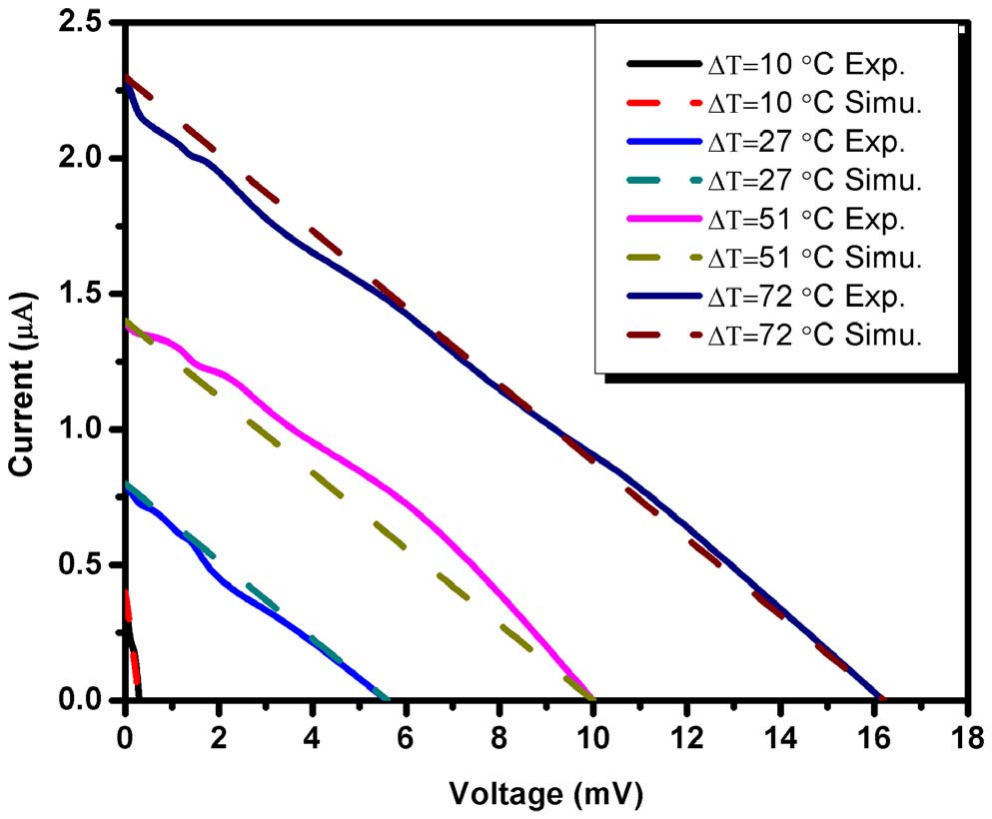
Comparison of experimental and simulated results of current-voltage behavior of the cells.

The modified form of above relationship for the current-voltage is the following
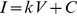
(6)where I is the short circuit current, V is open circuit voltage and k is current-voltage factor. The value of k for the data presented in Fig. 3 is calculated as −1.4×10^−4^ A/V. The comparison of experimental and simulated results is given in Fig. 6 and it is evident from the graphs that the experimental and simulated results are in good agreement.

For the simulation of open-circuit voltage and short circuit current-temperature gradient relationships the following exponential function is used [30]:
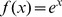
(7)


**Figure 7:**
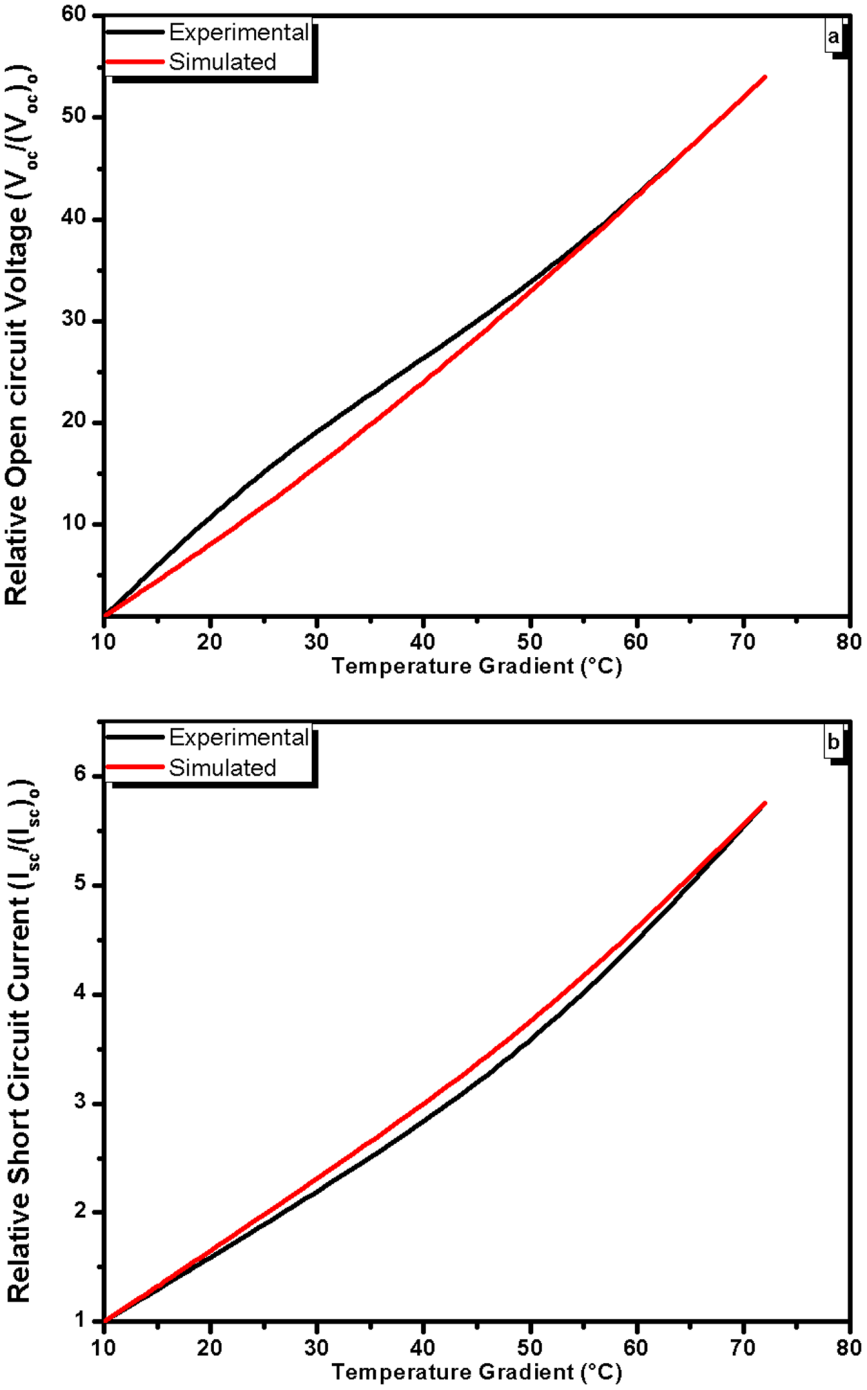
Experimental and simulated results of voltage-temperature gradient (a) and current-temperature gradient (b) behavior of the cells.

The Eq.4 has been modified for open circuit voltage-temperature gradient relationship as follows:
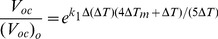
(8)where *(V_oc_)_o_* is initial open circuit voltage at minimal temperature gradient (ΔT = 10°C), *V_oc_* is open circuit voltage at instantaneous temperature gradients (*ΔT*) and k_1_ is voltage-temperature gradient factor. The value of voltage-temperature gradient factor is 6.4×10^−2^/°C. For the simulation of short circuit current-temperature gradient relationship the Eq.7 is modified as
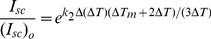
(9)where *(I_sc_)_o_* and *I_sc_* are initial short circuit current at minimal temperature gradient (ΔT = 10°C) and instantaneous values of short circuit current, respectively, while *ΔT* and *ΔT_m_* are temperature gradient and maximum temperature gradient accordingly. The k_2_ is current-temperature gradient factor and its value is calculated as is 2.8×10^−2^/°C. The comparison of experimental and simulated results of voltage-temperature gradient and current-temperature gradient relationship is given in Fig. 7(a) and Fig. 7(b), respectively. It is evident from the graphs that the simulated results are in agreement with experimental results. The further improvement in the simulation is possible, which may be carried out by our group as a future work.

## Conclusions

The investigation of the thermoelectric cells based on composites of carbon nano-tubes (CNT) and silicone adhesive showed that the cells have good stability of performance at large temperature gradient as well. It may allow the fabrication of less energy consuming (from the heater) temperature gradient sensors that can be used in instrumentation for the measurement of temperature gradient or for the generation of electric energy for low power applications. Moreover the efficiency of the cells can be increased by the modification of composites. The simulated results are in good agreement with experimental results.
